# Persistent Acute Onset Macroglossia Treated with Compression Therapy

**DOI:** 10.1155/2017/6402413

**Published:** 2017-09-11

**Authors:** Sean M. Johnson, C. Scott Brown, Liana Puscas

**Affiliations:** Division of Head and Neck Surgery and Communication Sciences, Duke University Medical Center, 40 Medicine Circle, M150, Green Zone, DUMC 2824, Durham, NC 27710, USA

## Abstract

Acute macroglossia, while rare and often limited in duration, can present significant management challenges. The anatomic position of the tongue, which can result in airway compromise in cases of enlargement, contributes significantly to difficulty with management. We review several management options for persistent acute onset lingual macroglossia and present a novel noninvasive management technique in a case which was refractory to several strategies.

## 1. Introduction

Acute onset macroglossia is well described within the literature as arising in numerous situations including patient positioning while under anesthesia, oropharyngeal packing, local trauma, postoperative from surgery in the oral cavity, or secondary to allergic or nonallergic angioedema [1–7]. Severe cases have the potential to present as an airway emergency due to oropharyngeal occlusion. Persistent cases can present significant management difficulties. Several management strategies have been described including utilization of reduction glossectomy [2, 4–6, 8, 9]. We present a rare case of severe lingual angioedema attributed to acquired C1 esterase inhibitor deficiency in the setting of systemic lupus erythematosus (SLE) which persisted despite utilization of previously described treatment strategies. A previously undescribed application of lingual compression wraps proved successful and is detailed.

## 2. Case Report

**Figure 1:**
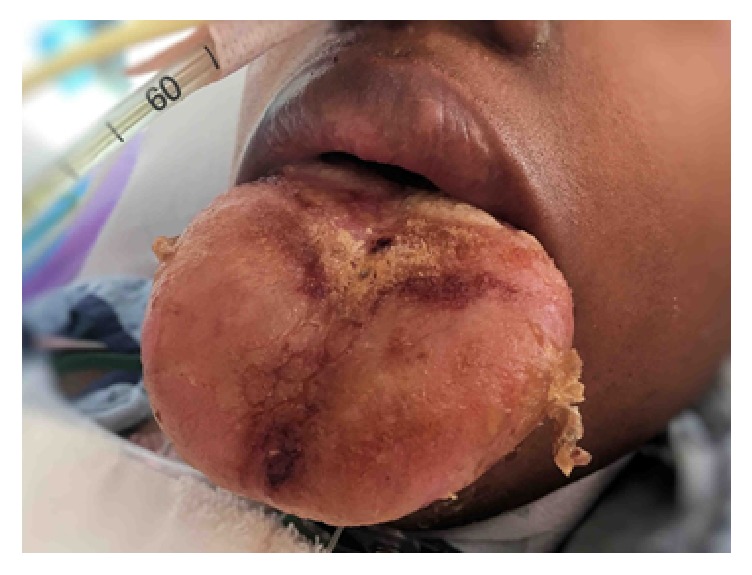
Lingual edema prior to initiation of lingual compression therapy. Note patient extubated with tracheostomy (not visible).

A 27-year-old female with a previous diagnosis of systemic lupus erythematous (SLE) was admitted to the hospital for management of acute on chronic renal failure diagnosed as lupus nephritis on biopsy. She developed acute respiratory failure on hospital day 18 requiring intubation and mechanical ventilation. Subsequent bronchoscopy supported a diagnosis of diffuse alveolar hemorrhage secondary to her SLE. Plasmapheresis was recommended by the consulting medical services in addition to her standard medical therapy. The patient developed severe acute onset angioedema of the face and oral cavity following completion of her second of five planned apheresis sessions. The consulting rheumatology service attributed the angioedema trigger to administration of fresh frozen plasma (FFP) as part of her apheresis that was later diagnosed as an acquired C1 esterase inhibitor deficiency secondary to SLE. Subsequently, albumin was substituted for FFP in her three remaining apheresis sessions without appreciated aggravation of her edema. Antihistamines were added to the IV Solu-Medrol she was receiving as part of her standard medical therapy. Resolution of her facial angioedema was noted with these conservative measures within a few days; however, her tongue remained severely edematous and protuberant (Figure 1).

Tracheostomy was completed seven days following onset of her angioedema given her persistent lingual edema and the anticipated prolonged course of mechanical ventilation. The otolaryngology service was contacted 16 days following surgery due to persistent tongue edema for consideration of possible partial glossectomy. Mild bite trauma was noted on the ventral aspect of the tongue but no significant trauma was noted dorsally. Her tongue was not able to be manually reduced. Bite blocks, fashioned out of silk tape and tongue depressors, were placed bilaterally between the premolars and molars at bedside. Vaseline gauze was then applied to the protruded tongue to help prevent desiccation.

**Figure 2:**
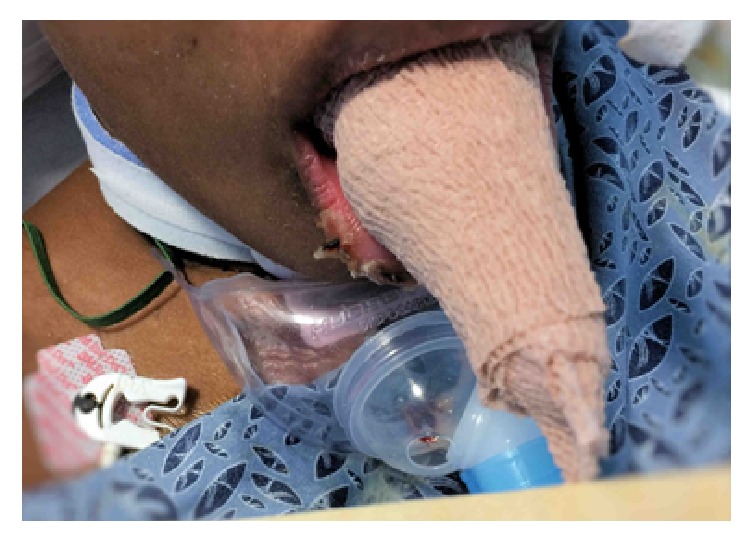
Lingual wrap in place. Self-adherent wrap visible.

No improvement in her lingual edema was noted after three days despite placement of the bite blocks. A lingual compression wrap was devised and initiated in an effort to avoid the need for partial glossectomy. First, a light wrap of moistened Kerlix was wrapped around the tongue from the distal tip proximally to the teeth. This was followed with a layer of self-adherent wrap also applied from the tip proximally to the teeth under light tension. A second pass of self-adherent wrap was then added with increased pressure titrated to patient tolerance (Figure 2). The wrap was replaced once a day and left in place for 12 hours at a time to allow direct inspection of the tissue and help mitigate potential pressure injuries. Significant improvement in the macroglossia was noted after only the first wrap and by day four the tongue was able to be reduced completely within the oral cavity with minimal effort. At this point, the compression wraps were discontinued and the bite blocks continued for two more days until the tongue was able to be retracted into the oral cavity by the patient without external effort. No evidence of lingual trauma secondary to the wraps was observed. The patient's respiratory status eventually recovered and she was decannulated without incident. She was noted with no long-term lingual sequela on subsequent outpatient follow-up visits several months later.

## 3. Discussion

Acute onset macroglossia can present significant management challenges when refractory to usual conservative therapy. If not already in use, IV glucocorticoids are a common initial step to address edema. Potential areas of mechanical lingual compression are then addressed. This point is emphasized by reports documenting massive lingual edema from placement of throat packs during surgery or even from endotracheal tubes alone [2, 4, 5]. Tracheostomy may be necessary to relieve compression of the tongue base in cases where intubation is prolonged.

Several previously described methods for treatment have been reported with success in resolving lingual edema. Saah et al. describe a case of traumatic macroglossia secondary to bite trauma in which edema did not resolve even after placement of tracheostomy and several days of IV steroids. Resolution of the edema was attained after only 48 hours following manual reduction of the edematous tongue. The reduction was maintained through use of a gauze headwrap keeping the mouth shut. The authors theorized that reduction of the tongue relieved lymphovascular obstruction [6]. The use of maxillary mandibular fixation following manual reduction was recently described by Shanti et al., also in a case of traumatic macroglossia. Fixation was maintained for three weeks at the end of which complete resolution of edema was noted [9]. In both cases a tracheostomy was preformed prior to utilization of these techniques.

In a case where the severity of lingual edema likely would not allow for reduction, Foreman et al. utilized bite blocks placed under anesthesia. In that case, extrinsic compression of the tongue by the teeth was suspected to be the primary factor in the persistence of edema. The patient was noted to tolerate bite blocks well and resolution of the edema was noted within a few days with no long-term morbidity [8].

The present case illustrates several of these management strategies. Our patient's initial presentation of lingual angioedema, later diagnosed as secondary to C1 esterase inhibitor deficiency, has been previously described in the literature [10]. Her facial edema resolved shortly after onset following administration of antihistamines and removal of the agent thought to trigger the episode, but her lingual edema persisted. Had our patient carried a known diagnosis of C1 esterase inhibitor deficiency, then prompt administration of a commercially available C1 esterase inhibitor may have adequately addressed her edema. However, this diagnosis was only made a few days following onset of the edema. Glucocorticoids were already in use for her SLE related renal disease and did not appear to alter her lingual swelling. The patient had a tracheostomy preformed due to lack of improvement of the lingual edema and the inability to extubate. The severity of her macroglossia proved too severe for manual reduction and a several-day trial of bite blocks, similar to that described by Foreman et al., proved unsuccessful. The application of a simple lingual wrap proved effective in inducing complete regression of the edema within a few days preventing the need for a morbid reduction glossectomy.

## 4. Conclusion

Acute onset macroglossia can be a dramatic and potentially life-threatening condition. Frequently, the condition is self-limited and resolves promptly. Persistent cases can present significant management challenges due to the need to maintain an adequate airway. Conservative management techniques often prove effective and the lingual compression wrap technique may prove successful in cases where other techniques have failed. Reduction glossectomy should be reserved as an option of last resort.
